# Noisy network attractor models for transitions between EEG microstates

**DOI:** 10.1186/s13408-020-00100-0

**Published:** 2021-01-04

**Authors:** Jennifer Creaser, Peter Ashwin, Claire Postlethwaite, Juliane Britz

**Affiliations:** 1 Department of Mathematics and EPSRC Centre for Predictive Modelling in Healthcare, University of Exeter, Exeter, UK; 2 Department of Mathematics, University of Auckland, Auckland, New Zealand; 3 Department of Psychology, University of Fribourg, Fribourg, Switzerland; 4 Neurology Unit, Medicine Section, Faculty of Science and Medicine, University of Fribourg, Fribourg, Switzerland

**Keywords:** EEG microstates, Excitable network model, Residence times, Transition process, Noisy network attractor, Long range temporal correlations

## Abstract

The brain is intrinsically organized into large-scale networks that constantly re-organize on multiple timescales, even when the brain is at rest. The timing of these dynamics is crucial for sensation, perception, cognition, and ultimately consciousness, but the underlying dynamics governing the constant reorganization and switching between networks are not yet well understood. Electroencephalogram (EEG) microstates are brief periods of stable scalp topography that have been identified as the electrophysiological correlate of functional magnetic resonance imaging defined resting-state networks. Spatiotemporal microstate sequences maintain high temporal resolution and have been shown to be scale-free with long-range temporal correlations. Previous attempts to model EEG microstate sequences have failed to capture this crucial property and so cannot fully capture the dynamics; this paper answers the call for more sophisticated modeling approaches. We present a dynamical model that exhibits a noisy network attractor between nodes that represent the microstates. Using an excitable network between four nodes, we can reproduce the transition probabilities between microstates but not the heavy tailed residence time distributions. We present two extensions to this model: first, an additional hidden node at each state; second, an additional layer that controls the switching frequency in the original network. Introducing either extension to the network gives the flexibility to capture these heavy tails. We compare the model generated sequences to microstate sequences from EEG data collected from healthy subjects at rest. For the first extension, we show that the hidden nodes ‘trap’ the trajectories allowing the control of residence times at each node. For the second extension, we show that two nodes in the controlling layer are sufficient to model the long residence times. Finally, we show that in addition to capturing the residence time distributions and transition probabilities of the sequences, these two models capture additional properties of the sequences including having interspersed long and short residence times and long range temporal correlations in line with the data as measured by the Hurst exponent.

## Introduction

The human brain is intrinsically organized into large-scale networks that can be identified when the brain is at rest [1, 2]. These networks reorganize on a sub-second temporal scale in order to allow the precise execution of mental processes [3]. Study of the spatial and temporal aspects of the dynamics underlying this reorganization requires non-invasive measures with high temporal resolution. The electroencephalography (EEG) is a direct measure of neuronal activity which captures the temporal evolution of the scalp electrical field with millisecond resolution. Unlike local measures of EEG in channel space that vary from time-point to time-point and as a function of the reference, the global measure of EEG topography remains stable for brief periods (50–100 ms) before changing to another quasi-stable state, the so-called EEG microstates [4, 5]. Microstates have been coined the “atoms of thought” and can be considered the basic building blocks of mentation that make up the spontaneous electrophysiological activity measured at the scalp [6].

At rest, when the subject is not doing any task, four dominant topographies are consistently reported both in healthy individuals across the lifespan as well as in neurological and psychiatric populations [7–12]. Moreover, neurological and psychiatric diseases have been shown to fundamentally alter their temporal dynamics [11, 13, 14]. This implies that the timing of the microstates and not the topography is the most crucial feature. Further evidence for the importance of the timing of the microstates comes from studies using simultaneously recorded EEG and functional magnetic resonance imaging (fMRI) that identify EEG microstates as the electrophysiological correlate of fMRI-defined resting-state networks (RSNs) [15–17]. This link is surprising because EEG microstates and fMRI-RSNs are two global measures of large-scale brain activity that are observed at temporal scales two orders of magnitude apart: 50–100 ms (microstates) and 10–20 seconds (fMRI). This link could only be established because EEG microstate time-series are scale-free, i.e., they do not occur at a characteristic timescale, but show similar behavior across different temporal scales. More specifically, EEG microstate sequences have been shown to have “memory”: they are mono-fractal and have long-range temporal correlations (LRTC) over six dyadic scales that span the two orders of magnitude (256 ms to 16 s) at which EEG microstate changes and fMRI blood-oxygen-level-dependent (BOLD) oscillations can be observed [18]. Gschwind *et al.* verify and expand the work of [18] by computing scale-free behavior in EEG data using a battery of tests, including computing the power spectral density and Hurst exponents using detrended fluctuation analysis (DFA), a wavelet framework, and time-variance analysis thus corroborating the robustness of the memory of microstate time series [19].

LRTC in microstate sequences suggest that the spatial-temporal dynamics are controlled not only by inherent structural properties of the fMRI-RSNs but also by a“sequential connectivity” that facilitates the timings and transitions between the underlying neural generators [20]. Importantly, Van de Ville *et al.* demonstrate that the precise timing of the residence times but not the order of local state transitions of the microstate sequences is crucial for their fractal properties [15]. Shuffling their state transitions without changing their timing has no effect, whereas equalizing their residence times degrades the time series to white noise. In the case that the residence times are IID and memoryless, then the residence times can be considered a “renewal process” and the whole process can be seen as Markov jump process. We note that the series of residence times and the state transition process are essentially independent. Therefore it is possible for the transitions to be Markov but the jump (residence) times to be non-Markov.

EEG microstate time series models must capture and reproduce four crucial aspects: (i) the local transition probabilities between states, (ii) the distribution of residence times, (iii) the long-range temporal correlations, and (iv) that longer and shorter residence times are interspersed throughout the sequence. So far, attempts to model EEG microstate sequences have been based on Markov-type models that assume the microstate time series is a memoryless process [21]. In particular, Gärtner *et al.* construct a hidden-Markov type stochastic model based on the transition probabilities between microstates extracted from the data [21]. This approach has been criticized for the underlying assumption that the microstate transition process is independent of the underlying global field power time series and therefore does not reproduce the LRTC [19, 22]. Von Wegner *et al.* show that neither memoryless Markov models nor single parameter LRTC models fully capture the data [23]. Hence, more sophisticated models need to be developed to capture the full spatiotemporal dynamics of microstate sequences.

In this paper we provide a novel modeling approach for microstate time series based on dynamical structures called *noisy network attractors*. These are stochastic models that exhibit *heteroclinic* or *excitable network attractors* in their noise-free dynamics [24]. A heteroclinic network is a collection of solutions (heteroclinic orbits) to a system of ordinary differential equations that link a set of steady states that themselves are unstable. Excitable networks, in the sense introduced in [24], are close relations of heteroclinic networks that have a small but finite threshold of perturbation that needs to be overcome to make a transition between a number of attracting states.

Heteroclinic networks have been found in models of many natural systems, for example, from population of neurons [25, 26], predator-prey dynamics [27], and bi-matrix game theory [28]. The dynamics near a network attractor is generally intermittent: trajectories spend long periods of time close to one state before switching to another. Heteroclinic cycles or networks have been successfully applied to model transient dynamics in neural processes at a variety of levels of description and spatial scales [29]. For example, from olfactory processing in the zebra fish [30] to human working memory [31], episodic memory [32], and decision making [33]. Time series from similar networks with noise have previously been shown to produce non-Markovian dynamics [24, 34]. These modeling techniques have also been used successfully in applications of artificial intelligence. For example, in [35] the authors show that arbitrary Turing machines can be built from these excitable networks, and in [36] the authors use networks of this form as the ‘brain’ for a theoretical robot that has to solve a classification task. Given the powerful capacities of network attractors to model transient dynamics at different levels, these are a promising candidate to model EEG microstate sequences.

Here we demonstrate that the transitions and LRTC of EEG microstates sequences can be modeled using stochastic differential equations (SDEs) that possess such an excitable network attractor. We show that the residence time distribution of the microstate time series follows a double exponential decay. We first construct a four-node, all-to-all connected network model. The SDEs we use have low amplitude additive white noise, and the network is designed using the construction outlined in [24]. Each node in the network represents one microstate, and the transitions between them are driven by the noise on the edges. This model captures the series of state transitions, but not the distribution of residence times. We present two extensions to this model that induce longer residence times observed in the data using two different mechanisms. The first is to add a hidden node for each microstate that acts as a trap for the transient dynamics. The second incorporates a controlling layer with two nodes representing a central switching control of faster and slower switches between the nodes or the original network. Finally, we assess the interspersion of long and short residence times by means of their autocorrelation and show that, when fitted to the data, the sequences generated with each extended model have the same extent of LRTC as measured by the Hurst exponent [37].

## EEG data collection and analysis

Detailed description of the procedures used to collect the EEG recordings and convert them into microstates are given in [18]; here we provide a brief summary for completeness. Nine healthy volunteers (24–33 years, mean age 28.37 years) participated for monetary compensation after giving informed consent approved by the ethics commission of the University Hospital of Geneva. None suffered from current or prior neurological or psychiatric illness or from claustrophobia. EEG was recorded from 64 sintered Ag/AgCL electrodes in an extended 10–10 system. EEG was digitized using a battery-powered and MRI-compatible EEG system (BrainAmp MR plus, Brainproducts) with a sampling frequency of 5 kHz and a hardware bandpass filter of 0.016–250 Hz with the midline fronto-central electrode as the physical reference. For each subject, we recorded one 5-minute session outside the scanner prior to recording three 5-minute runs of resting-state EEG inside the MRI scanner. Subjects were instructed to relax and rest with their eyes closed without falling asleep and to move as little as possible. Data from one subject had to be excluded due to subsequent self-report of sleep and the presence of sleep patterns in EEG. The 24 recordings from the remaining eight subjects were submitted for further processing. MRI and ballistocardiographic artifacts were removed using a sliding average, then oculomotor and myogenic artifacts were removed using independent component analysis. The data were downsampled to 125 Hz and bandpass filtered between 1 and 40 Hz.

**Figure 1:**
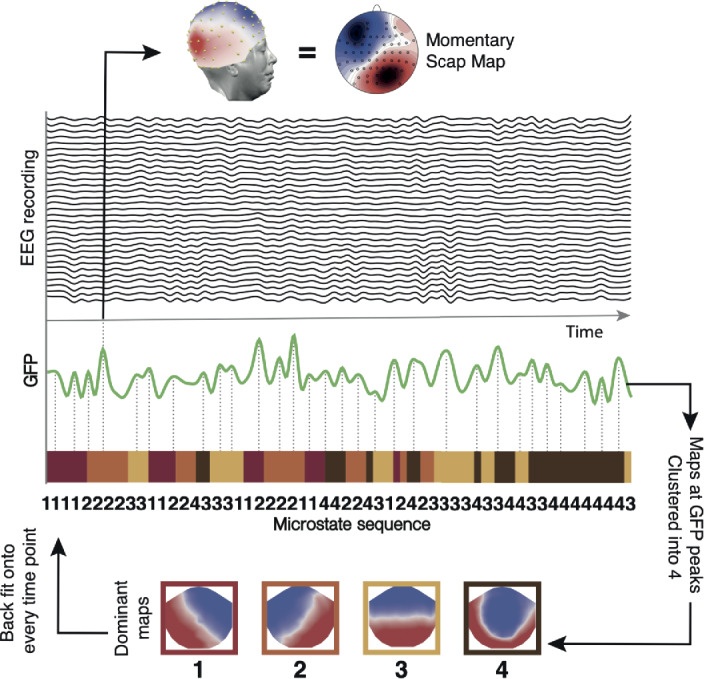
Schematic of the EEG microstate method. An example EEG recording and global field power (GFP, the spatial standard deviation of the electrical field). Momentary maps at the peaks of GFP are submitted to an AAHC clustering algorithm. The four dominant template maps are shown at the bottom. Backfitting is performed by calculating the spatial correlation between the four template maps and the continuous EEG data at each time point. Labels are assigned in a winner-take-all manner to generate the microstate sequence

To compute microstate sequences, first the global field power (GFP) was computed from each recording as the standard deviation of the scalp electrical field at each time point. Between the local troughs of GFP, the scalp topography remains stable and only varies in strength. EEG at all local peaks of GFP was extracted and submitted to a modified atomize-agglomerate hierarchical clustering (AAHC) algorithm [38]. Four dominant template maps were identified as the optimal solution in each run using a cross-validation criterion, a measure of residual variance [39]. These four dominant maps are in line with the classically identified microstates [5, 7, 12]. Finally, the microstates were fitted back onto EEG by computing the spatial correlation between the four template maps and the continuous EEG data, then the template that correlated highest with the data was assigned at each time point. Figure 1 shows a schematic of the EEG microstate procedure and the four dominant template maps. Note that at the local troughs of GFP the field strength is minimal and none of the template maps correlate well with the data; the allocation of the best template can be considered random. To avoid these brief periods being accounted as very short separate microstates, a temporal constraint criterion is required. We use a temporal constraint criterion of 32 ms (corresponding to four sample points at 125 Hz) to obtain a time-series of the dominant EEG microstates for each recording. Figure 10 in Appendix A illustrates the requirement for the temporal constraint.

The microstate time-series is a sequence that we denote by $m(t)$ where $m\in \{1,2,3,4\}$ at any given sampling time point *t*. We classify $m(t)$ into *epochs* of the same state defined by saying that we enter a new epoch at the time point that the microstate changes, if $m(t+1)\neq m(t)$; note that we enter a new epoch at the start of the sequence $t=0$. The average number of epochs per recording is 2010. We describe the *k*th epoch in terms of its *state* $\sigma (k)$ and *residence time* $\rho (k)$ respectively. We call the sequence of states visited $\sigma (k)$ the *transition process*, and the sequence of residence times $\rho (k)$ a *residence process*. These processes can, at least in principle, be independent. We define:

- $R(t)$ is the distribution of residence times $\rho (k)$ for all epochs *k* (regardless of state).
- $T(m,j)$ is the probability of transition from an epoch in state *m* to one in state *j*,
  $$ T(m,j)= \frac{\#\{k: \sigma (k)=m \text{ and }\sigma (k+1)=j\}}{\#\{k: \sigma (k)=m\}}. $$

Note that there are no self-transitions due to our definition of an epoch. To identify the residence distribution *R* from the data, we compute the histogram of residence times from each microstate sequence for bin size 40, then we find the average and standard error of each bin over all recordings. The distribution is truncated at the first empty bin. We compute the residence distribution *R* for the data collected inside and outside the scanner, then we use a two-sample Kolmogorov–Smirnov test and find that the histograms are from the same continuous distribution. We also calculate *T* for each recording and find the average and standard error for each probability over all recordings. Calculating the average *T* for the two groups, from inside and outside the scanner, separately we find no significant differences for any transition between the two groups using a Kruskall–Wallis test with Bonferroni correction. Therefore, we combine the recordings from inside and outside the scanner for subsequent analysis and model fitting.

To quantify the combined distribution from the data, we fit exponential curves given by

1 $$ {\mathcal{E}}^{(n)}(t)= \sum_{i=1}^{n}a_{i} \exp (-k_{i}t) $$

for $n=1,2,3$ to the data using Matlab, and constrain $a_{i}>0$ and $k_{i}\geq 0$. We also fit power law curves given by

2 $$ {\mathcal{P}}^{(1)}(t)= b_{1}t^{c_{1}},\qquad { \mathcal{P}}^{(2)}(t)= b_{2}t^{c_{2}} +d. $$

We calculate the error *χ* as the least squares difference between the log of the values of each curve and the log of the data. We use an F-test (code used as written for [40]) with threshold $\alpha =0.05$ to indicate if extra parameters are warranted to improve the fit of the data.

**Figure 2:**
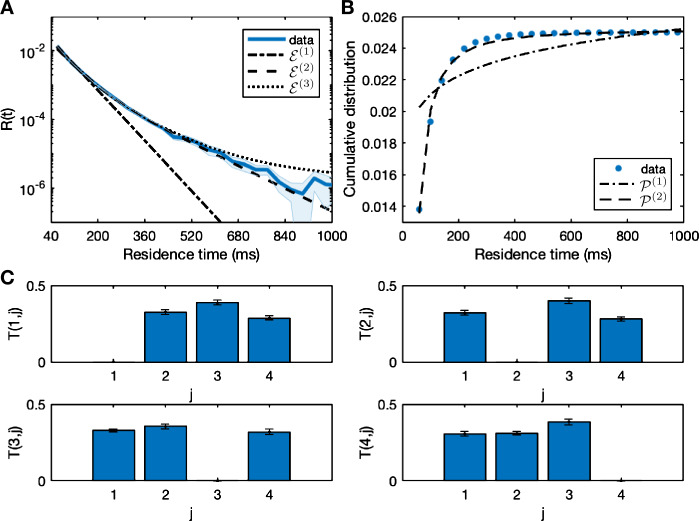
Transition probabilities and residence times extracted from the data. Panel (**A**) shows the residence time histogram for the data the scanner, with bin size 40. The curves for one-phase ${\mathcal{E}}^{(1)}$, two-phase ${\mathcal{E}}^{(2)}$, and three-phase decay ${\mathcal{E}}^{(3)}$ are shown. Panel (**B**) shows the cumulative residence times with power law curves ${\mathcal{P}}^{(1)}$ and ${\mathcal{P}}^{(2)}$. Curve details are in Table 1. The bottom four panels (**C**) show the probability of transition $T(m,j)$ from state *m* to state *j* for each microstate $m={1,2,3,4}$

The residence time distribution $R(t)$ and transition probabilities $T(m,j)$ from the data are shown in Fig. 2. The distribution $R(t)$ is plotted on a logarithmic scale with ${\mathcal{E}}^{(n)}(t)$ for $n=1,2,3$ given by (1) and ${\mathcal{P}}^{(n)}(t)$ for $n=1,2$ given by (2). The values of the coefficients are given in Table 1. We perform a comparison of ${\mathcal{E}}^{(1)}$ and ${\mathcal{E}}^{(2)}$ and find that $F(d_{n},d_{d}) = 1102.8(2,31)$, which therefore rejects the null hypothesis that the distribution is ${\mathcal{E}}^{(1)}$. We also compare ${\mathcal{E}}^{(3)}$ and ${\mathcal{E}}^{(2)}$ and find that $F(d_{n},d_{d}) =46.9(2,30)$, therefore the additional parameters are warranted. Figure 2 shows that although ${\mathcal{E}}^{(3)}$ has smaller error, the improvement in fit is for short residence times and the tail in this case is better captured by ${\mathcal{E}}^{(2)}$ which lies within the error bars for large residence time values. Figure 2 also shows the cumulative distribution of residence times and the two power law curves. We perform a comparison of ${\mathcal{P}}^{(1)}$ and ${\mathcal{P}}^{(2)}$. We find that an *F*-test rejects the null hypothesis that the distribution is ${\mathcal{P}}^{(1)}$, with $F (d_{n},d_{d}) = 1167 (2,32)$.

**Table 1:** The values for the constants for the exponential curves ${\mathcal{E}}^{(n)}$ for $n=1,2,3$ given by (1) and the power law curves ${\mathcal{P}}^{(n)}$ for $n=1,2$ given by (2). The distance *χ* from the data is given for each curve

	${\mathcal{E}}^{(1)}$	${\mathcal{E}}^{(2)}$	${\mathcal{E}}^{(3)}$		${\mathcal{P}}^{(1)}$	${\mathcal{P}}^{(2)}$
$a_{1}$	4.875 × 10^−2^	3.451 × 10^−2^	3.766 × 10^−2^	$b_{1}$	1.473 × 10^−2^	
$k_{1}$	2.114 × 10^−2^	2.087 × 10^−2^	2.113 × 10^−2^	$c_{1}$	7.79 × 10^−2^	
$a_{2}$		2.829 × 10^−3^	2.432 × 10^−3^	$b_{2}$		−8.205
$k_{2}$		9.510 × 10^−3^	9.293 × 10^−3^	$c_{2}$		−1.602
$a_{3}$			1.052 × 10^−5^	*d*		2.519 × 10^−2^
$k_{3} $			1.405 × 10^−3^			
*χ*	697.6697	9.9775	2.4163		0.2005	0.0027

We note that the error in the tail of the distribution is relatively large. There are few very long residence times $840<\rho <1000$ ms, and longer recordings would be required for greater certainty about the distribution of the very long (>900 ms) residence times. The heavy tailed residence time distribution indicates non-Markovian dynamics in line with [19]. This is supported by the best fit of the power law ${\mathcal{P}}^{(2)}$ with a negative slope that is synonymous with scale-free dynamics [41]. When fitting the models to the data, we will consider residence times $20<\rho <900$ ms due to the greater uncertainty for >900 ms; details of the fitting strategy are given in each section.

## Single-layer excitable network model

We aim to build a model that captures the statistical properties of the transition process and residence process. To this end, we construct a model of stochastic differential equations perturbed by low amplitude additive white noise, using a general method that allows us to realize any desired graph as an attracting excitable network in phase space. This model has evolved from work in [42] and is detailed in [24], and here we briefly outline the construction.

We realize the network of all possible transitions between the four canonical microstates as an excitable network with four nodes (*p*-cells) that represent the four microstates. There is an edge between two nodes in the network if there can be transition between the two corresponding microstates; here the network is all-to-all connected with twelve edges (*y*-cells).

The system is given by

3 $$\begin{aligned} & \tau \,\mathrm {d}p_{j} = \bigl[ f(p_{j},y_{k}) \bigr]\,\mathrm{d}t + \eta _{p} \,\mathrm {d}w_{j}, \end{aligned}$$

4 $$\begin{aligned} & \tau \,\mathrm {d}y_{k} = \bigl[g(p_{j},y_{k}) \bigr] \,\mathrm {d}t + \eta _{{y}_{k}} \,\mathrm {d}w'_{k} \end{aligned}$$

for $j=1,\dots,M$ and $k=1,\dots,Q$. We create a microstate model by choosing $M=4$ (corresponding to the number of states) and $Q=12$ (corresponding to the number of possible transitions between states). The $w_{j}$ and $w_{k}'$ are independent identically distributed (iid.) noise processes, and *η* is noise weights. We introduce the time scaling *τ* (which does not occur in [24]) because although the timescale of the *p*-dynamics can be scaled by the parameters, the equations for the *y* dynamics have a functional form in which the timescale is fixed. The $p_{j}$ variables classify which node of the network (i.e., which of the four microstates) is visited. The $y_{k}$ variables become non-zero during a transition between nodes. The functions *f* and *g* and parameters for this single-layer model are detailed in [24] and discussed in Appendix B.1.

This network can be realized as either a heteroclinic or excitable network depending on the choice of constants in *f* and *g* [24]. In the system with no noise, the network consists of heteroclinic connections between equilibrium solutions with $p_{j}=1$ and all other coordinates zero. In an excitable network, these equilibria are all stable, and small perturbations (from added noise) are required to push the trajectory from one equilibrium to another.

**Figure 3:**
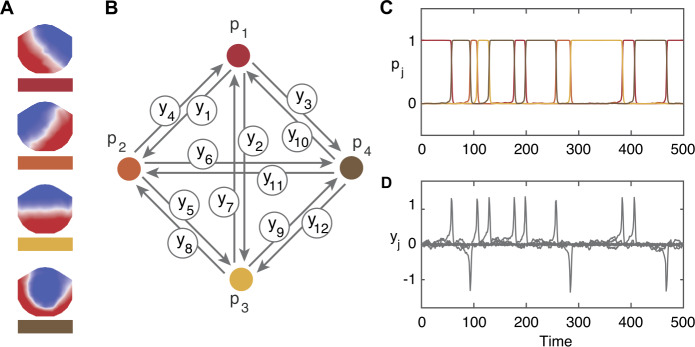
Structure and dynamics of the excitable network model. Panel (**A**) shows the four dominant microstates. Panel (**B**) shows the coupling architecture of the sixteen-cell network. Each node represents one of the microstates shown in panel (**A**). Each edge represents allowed transitions between microstates. Panel (**C**) shows the time series of the *p*-cells (nodes), note at most only one node is equal to one at any given time point. Panel (**D**) shows the time series of the *y*-cells (edges). The edges only become active (non-zero) during transitions between nodes

Figure 3 shows the four microstates from the data and the coupling architecture of the network model with example time series output for the nodes and the edges in the excitable case. The transitions between nodes occur on a faster timescale than the length of time spent at each node (the residence time). Thus, the trajectory in the simulation is almost always close to one of the equilibrium solutions, where one of the $p_{m}$ variables is close to 1.

To determine when the trajectory is close to a given node, we define a box in phase space around that node so that when the trajectory is in the box we say it is near the node. Here we fix the box size $h= 0.49$ such that each box contains one node and the boxes do not overlap. The duration of time spent in the box is then the residence time for that node [24]. The output of the model simulation is a series of *k* epochs where each epoch is defined by the state it is in $\sigma \in \{1,2,3,4\}$ and its residence time *ρ*. The transition rates between nodes in the model are modulated by the noise levels on the edges (rather than on the nodes) as described in [24]. Therefore we fix the noise on the nodes $\eta _{p}=10^{-4}$. The free parameters for fitting the model are the twelve noise values on the edges $\eta _{y_{k}} k=1,\dots,12$.

To fit the excitable network model output to the data, we employ the following algorithm.

1. *Initialize*: Initiate the noise values using scaled transition probabilities $\eta ^{0}_{y_{k}} = {\mathcal{S}}^{0}n^{0}_{k}$, where $n^{0}_{k}=T(m,j)$ and ${\mathcal{S}}^{0}=1$.
2. *Model simulation and analysis*: Numerically simulate the excitable network model given by system (3)–(4) with a stochastic Heun method implemented using a custom code written in Matlab. Compute ten realizations of the model using step size 0.05 up to maximum of 100,000 steps. This gives approximately 5750 epochs per realization. Calculate the transition probabilities $T_{s}(m,j)$ and residence distribution $R_{s}(t)$ from the simulations in the same way as from the data; see Sect. 2.
3. *Cost function*: Compute the cost function
  5 $$ {\mathcal{C}} = \sum_{t}\bigl( \bigl\vert \log \bigl(R(t)\bigr) \bigr\vert - \bigl\vert \log \bigl(R_{s}(t)\bigr) \bigr\vert \bigr)^{2} + 100\sum_{m,j} \bigl(T(m,j)-T_{s}(m,j)\bigr). $$
  This function is the weighted sum of the distance between the log of the residence times $R(t)$ (from the data) and $R_{s}$, and the distance between the transition probabilities *T* and $T_{s}$. The weighting for the transition probabilities is due to the relatively smaller magnitude of the difference. We seek to minimize this cost function.
4. *Update parameters*: Change ${\mathcal{S}}^{i}$ and $n^{i}_{k}$ according to the following strategy. Change $n^{i}_{k}$ values to minimize the least square distance between the transition probabilities *T* and $T_{s}$. Maintain the ordering between the probabilities for a given m, for example, if $T(1,3) > T(1,2)$ then we set $n^{i}_{2}>n^{i}_{1}$. In this way the excitable network model can be adjusted to produce any given set of transition probabilities, as described in [24]. Change ${\mathcal{S}}^{i}$ to control the decay rate of $R_{s}$ and minimize the least squares distance between the log residence times *R* and $R_{s}$. If all $\eta _{y_{k}}$ are large, for example, $O(10^{-1})$, transitions happen quickly, the residence times are short, and the decay rate of the distribution *R* is large (slope is steep); whereas if $\eta _{y_{k}}$ are small, for example, $O(10^{-4})$, there are long residence times between transitions and the decay rate is small (slope is shallow). Set $\eta ^{i}_{y_{k}} = {\mathcal{S}}^{i}n^{i}_{k}$.
5. Repeat from 2.

**Figure 4:**
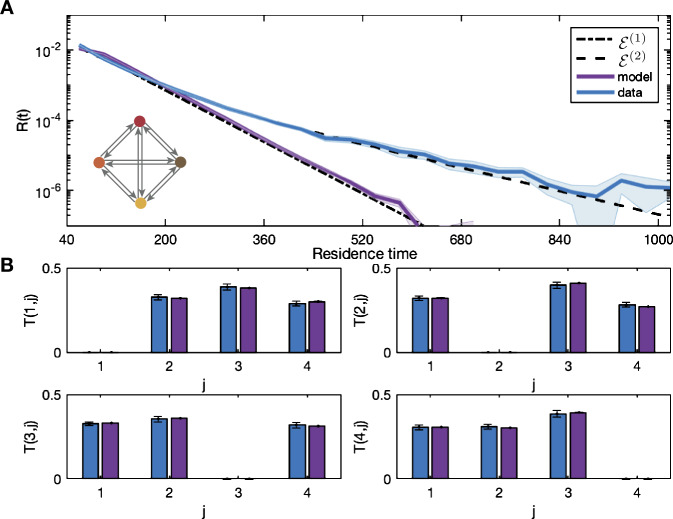
A four-state excitable network model that captures transition probabilities but not temporal dynamics. Excitable network model simulation (purple) with residence distribution for the data (blue) in panel (**A**) with inset network schematic. Curves ${\mathcal{E}}^{(1)}$ and ${\mathcal{E}}^{(2)}$ from Table 1 are also shown (black dashed lines). The transition probabilities $T(m,j)$ are shown in panels (**B**) for both the data (blue) and the simulation (purple). The parameters used in the simulations and the cost function are given in Table 2. There is good agreement between the model transition probabilities, and the residence time distribution of the model closely follows the single exponential curve rather than the data distribution

The statistics of the sequences generated by the excitable network model to the data are shown in Fig. 4 with the curves ${\mathcal{E}}^{(1)}$ and ${\mathcal{E}}^{(2)}$ from Table 1. The noise weights $\eta _{y_{k}}$ used here and corresponding cost function ${\mathcal{C}}$ are given in Table 2. Our aim is to demonstrate whether this model can produce the statistical properties of interest, and so adapting the parameters at each iteration is performed in an ad-hoc manner according to the fitting strategy. In this case the fitting algorithm is stopped when the model transition probabilities lie within the error bars of the data and residence distribution is aligned with the single exponential curve ${\mathcal{E}}^{(1)}$. Here, no choice of $\eta _{y_{k}}$ allows the distribution to follow ${\mathcal{E}}^{(2)}$ as the transition process generated by the model is Markov and the residence times correspond to inter-event intervals for a Poisson process [24]. This is further evidence that this modeling approach cannot produce a heavy tailed residence distribution observed in the data. Due to the evidence of multiscale behavior of the temporal dynamics of microstate sequences [18, 19], we now present two extensions of the model, each of which generates a residence process distribution with a heavy tail.

**Table 2:** Parameters for the excitable network model. The values $\eta _{y_{k}}$ identified following fitting to the data shown in Fig. 4. Here ${\mathcal{S}}=0.1$ and the cost function ${\mathcal{C}} = 106.954$

$\eta _{y_{1}}$	$\eta _{y_{2}}$	$\eta _{y_{3}}$	$\eta _{y_{4}}$	$\eta _{y_{5}}$	$\eta _{y_{6}}$
0.0334	0.0355	0.0326	0.0347	0.0379	0.0328
$\eta _{y_{7}}$	$\eta _{y_{8}}$	$\eta _{y_{9}}$	$\eta _{y_{10}}$	$\eta _{y_{11}}$	$\eta _{y_{12}}$
0.0318	0.0328	0.0312	0.0335	0.0334	0.0366

## Hidden-node network model

The excitable network model was constructed using a general method that allows us to realize any desired graph as an attracting excitable network in phase space. We can use the same method of construction to extend the network model to a network containing 8 nodes and 20 edges (28 cell network) such that each node in the original four-node network is connected to one additional “hidden node”. Specifically, we use the system of SDEs given by (3)–(4) with $M=8$ and $Q=20$. We then modify the outputs $(O)$ and inputs $(I)$ to the *p* cells from the *y* cells so that each of the original four nodes has one additional node bidirectionally coupled to it. We introduce noise weights $y_{\mathrm{{out}}}$ and $y_{\mathrm{{in}}}$ on the edges connecting the hidden nodes to the network. They drive transitions out to the hidden nodes and back again, respectively.

**Figure 5:**
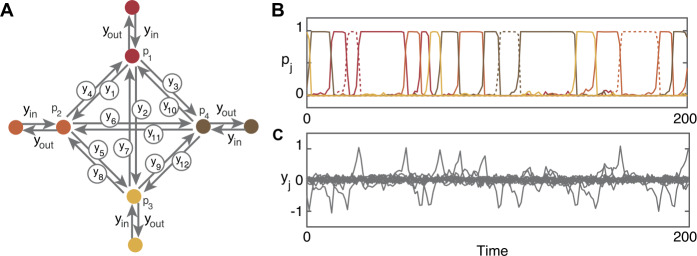
Structure and dynamics of the hidden-node network model. Panel (**A**) shows the coupling architecture of the 28-cell network consisting of the network of four nodes representing the four microstates with one hidden node for each microstate. Note that $y_{\mathrm{{out}}}$ and $y_{\mathrm{{in}}}$ are the same for all hidden nodes. Panel (**B**) shows the time series of the eight *p*-cells (nodes), note at most only one node is equal to 1 at any given time point. The solid lines are the trajectories of the original four $p_{i}$ cells (as shown in Fig. 3) and the dotted lines are the trajectories of the additional nodes, colored according to the $p_{i}$ they are attached to. Note here that when a hidden node is equal to 1 it contributes to the residence time of its neighboring node. Panel (**C**) shows the time series of the 20 *y*-cells (edges). The model was simulated using exemplar noise values of $\eta =0.05$ on all edges

Figure 5 shows the four-node network from Fig. 3 with the additional four hidden nodes and example time series output for the nodes and the edges. Again, at almost all points in time, the trajectory is close to one of the equilibrium solutions; when the trajectory is close to a hidden node, we record the output as being at the node neighboring that particular hidden node. The transition probabilities are independent of how long it is spent at the hidden node.

The constants in *f* and *g* are fixed as in Sect. 3, so the whole network is excitable and transitions are driven by noise. We fix the noise value on all nodes $\eta _{p}=10^{-4}$ as before. The free parameters for fitting the model are the twelve noise weights on the edges $\eta _{y_{k}}$ and the two additional noise weights $y_{\mathrm{{out}}}$ and $y_{\mathrm{{in}}}$. To fit the model to the data, we use a similar procedure to that described in Sect. 3.

1. *Initialize*: Set $\eta ^{i}_{y_{k}} ={\mathcal{S}}^{0} \eta ^{0}_{y_{k}}$ using the values from Table 2 and $y_{\mathrm{out}}=y_{\mathrm{in}}=0.1$.
2. *Model simulation and analysis*: Simulate the hidden-node model and calculate the transition probabilities $T_{s}(m,j)$ and residence distribution $R_{s}(t)$ as described in Sect. 3.
3. *Cost function*: Compute the cost function ${\mathcal{C}}$ using (5).
4. *Update parameters*: For this model the shortest residence times are captured by transitions around the original four nodes. Set ${\mathcal{S}}^{i}$ to produce shorter residence times, then update $n_{k}^{i}$ as described in Sect. 3. The longer times are achieved by visits to the hidden nodes. Set $\eta _{y_{\mathrm{{out}}}}$ to control how often the hidden nodes are visited, for example, if $\eta _{y_{\mathrm{{out}}}}<\min (\eta _{y_{k}})$, the probability of visiting a hidden node is less than visiting a neighboring node. Set $\eta _{y_{\mathrm{{in}}}}$ to control how long it is spent at the hidden node; if $\eta _{y_{\mathrm{{in}}}}$ is large, then the time at the hidden node will be shorter than if $\eta _{y_{\mathrm{{in}}}}$ is small.
5. Repeat from 2.

**Figure 6:**
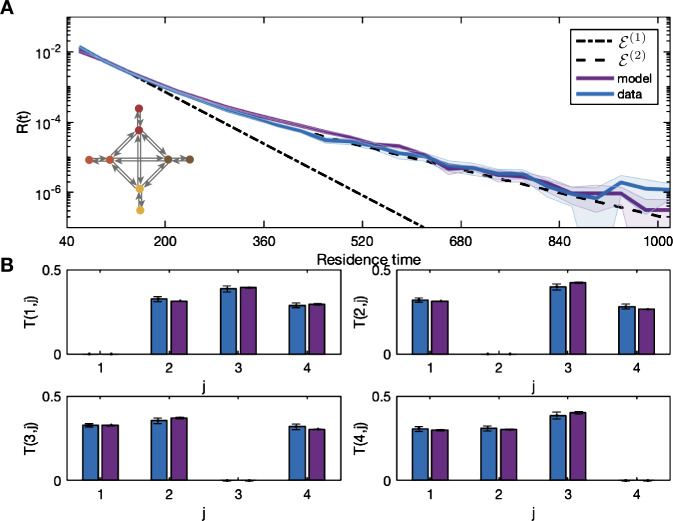
Hidden-node network model captures transition probabilities and temporal dynamics. Hidden-node network model simulation (purple) with residence distribution for the dataset (blue) in panel (**A**) with inset network schematic. The single and double exponential curves from Table 1 are shown for comparison (black dashed lines). The transition probabilities $T(m,j)$ are shown in panels (**B**) for both the data (blue) and the simulation (purple). The parameters used in the simulations in each panel are given in Table 3. There is good agreement between the model transition probabilities; compare with Fig. 4. The residence time distribution of the hidden-node model closely follows the double exponential curve and the distribution of the data

The results of fitting the hidden-node network model with four hidden nodes to the data are shown in Fig. 6. For illustration we also show the exponential curves ${\mathcal{E}}^{(n)}$ for $n=1,2$ from Table 1. The noise weights $\eta _{y_{k}}$ used and cost function are given in Table 3. The noise weights $\eta _{y_{\mathrm{{in}}}}$ and $\eta _{y_{\mathrm{{out}}}}$ are lower than the noise weights $\eta _{y_{k}}$. This means that the trajectory is more likely to transition to another microstate node than to the hidden node, and when it is at a hidden node it takes longer to transfer to another node, i.e., it gets trapped. This gives longer residence times for each node than the four-node network alone.

**Table 3:** Parameters for the hidden-node network model. The noise values on the edges $\eta _{y_{k}}$ and the additional edges $\eta _{y_{\mathrm{{out}}}}$ and $\eta _{y_{\mathrm{{in}}}}$ for the model simulation to fit the twelve transition probabilities of the data shown in Fig. 6. Here ${\mathcal{S}} = 0.17$ and ${\mathcal{C}} = 1.268$

$\eta _{y_{\mathrm{{out}}}}$	$\eta _{y_{\mathrm{{in}}}}$				
0.032	0.052				
$\eta _{y_{1}}$	$\eta _{y_{2}}$	$\eta _{y_{3}}$	$\eta _{y_{4}}$	$\eta _{y_{5}}$	$\eta _{y_{6}}$
0.0501	0.0533	0.0489	0.0521	0.0567	0.0494
$\eta _{y_{7}}$	$\eta _{y_{8}}$	$\eta _{y_{9}}$	$\eta _{y_{10}}$	$\eta _{y_{11}}$	$\eta _{y_{12}}$
0.0477	0.0492	0.0468	0.0503	0.0501	0.0548

This model allows local control at each state. However, it is not easily expandable to accommodate additional decay rates or external influences, for example, from other systems in the body. We therefore also present a more parsimonious alternative, namely a more generalizable model in which the distribution of residence times is not controlled independently at each node but by an additional controlling layer.

## Multi-layer network model

We present a second extension to the excitable network model by including a controlling layer that drives the transitions around the original four-node network. To this end, we construct a system of *N* levels for which we set up a system of SDEs as follows:

6 $$\begin{aligned} &\tau \,\mathrm {d}p_{l,j} = \bigl[f_{l}(p_{l,j},y_{l,k}) \bigr] \,\mathrm {d}t+\eta _{p} \,\mathrm {d}w , \end{aligned}$$

7 $$\begin{aligned} &\tau \,\mathrm {d}y_{l,k} = \bigl[g_{l}(p_{l,j},y_{l,k})+ z_{l,k} \bigr] \,\mathrm {d}t+\eta _{{l,k}} \,\mathrm {d}w_{k}. \end{aligned}$$

Each level *l* has $M_{l}$ nodes, and we assume all-to-all connections in each layer, so we have $Q_{l}\equiv M_{l}(M_{l}-1)$ edges. *w* is independent identically distributed noise processes, *η* is noise weights. This system includes a general input into the *y*-cells $z_{l,k}(t)$ that linearly couples layers from the $p_{l}$ nodes to the $y_{l+1,k}$ edges by

$$ z_{l+1,k}= \sum_{j=1}^{M_{l}}\zeta _{l,j} p_{l,j}^{2}. $$

The functions *f* and *g* and parameters for this hidden-layer model are detailed in [24] and discussed in Appendix B.2.

For the microstate model, we choose the number of levels $N=2$, the number of nodes in layer one $M_{1}=2$, in layer two $M_{2}=4$, the number of edges in layer one $Q_{1}=2$ and in layer two $Q_{2}=12$. The constants in functions *f* and *g* are set as in Sect. 3 for each layer, so each layer is an excitable network. Note that layer two is identical to the excitable model described in Sect. 3.

**Figure 7:**
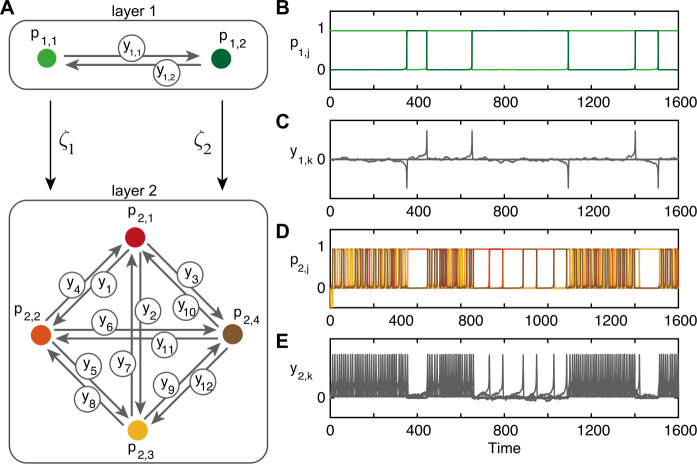
Structure and dynamics of a multi-layer model. Panel (**A**) shows the coupling architecture of the two networks; compare layer two to Fig. 3(B). Example time series are shown for layer one nodes in panel (**B**) and edges in panel (**C**), layer two nodes in panel (**D**) and edges in panel (**E**). The noise on all edges $\eta _{l,k}=10^{-2}$ is fixed, residence times are scaled by $\zeta _{1} = 10^{-1}$ and $\zeta _{2} =10^{-3}$. When $p_{1,2}=1$ in panel (**A**), the dynamics on the edges in layer two are scaled by $\zeta _{2}$ and residence times of nodes in layer two, shown in panel (**D**), are much longer than when $p_{1,1}=1$

Layer one with two nodes is a “controlling layer” in that we only consider the output from layer two with four nodes. As before the output is a transition process and a residence process. The two nodes in layer one affect the dynamics on the edges (and therefore the residence times) in layer two by the scalings $\zeta _{1,1}=\zeta _{1}$ and $\zeta _{1,2}=\zeta _{2}$. These scale the residence times in the output from layer two. As before we fix the noise on the nodes $\eta _{p}=10^{-4}$. Figure 7 shows the coupling architecture for the two-layer model with time series of the nodes and edges in each layer. Note that layer two is the same as in Fig. 3(B). For illustrative purposes, in Fig. 7 we choose $\zeta _{1}=10^{-1}$ and $\zeta _{2}=10^{-3}$ here. Panels (B)–(E) clearly show that when $p_{1,2}=1$ in layer one the residence times at each node in layer two $p_{2,j}$ are longer (transitions between nodes are less frequent) as the edges in layer two $y_{2,k}$ are scaled by $\zeta _{2}$. Note that if $\zeta _{1}=\zeta _{2}$ layer one would be redundant as the residence times would be consistent (drawn from the same distribution) in either node and the residence process would again be exponential.

The free parameters for fitting the model are the two noise values on the edges in layer one $\eta _{1,k}$ for $k=1,2$, the 12 noise values on the edges in layer two $\eta _{2,k}$ for $k=1,\dots,12$, and the two transfer parameters $\zeta _{l}$ for $l=1,2$. To fit the multi-layer model, we employ the fitting algorithm as for the other two models.

1. *Initialize*: Set $\eta ^{i}_{2,k}$ using the values from Table 2. Set $\eta _{1,k}=10^{-2}$, $\zeta _{1}=10^{-1}$, and $\zeta _{2}=10^{-3}$.
2. *Model simulation and analysis*: Simulate the multi-layer model and calculate the transition probabilities $T_{s}(m,j)$ and residence distribution $R_{s}(t)$, as described in Sect. 3, from the output of layer two only.
3. *Cost function*: Compute the cost function ${\mathcal{C}}$ using (5).
4. *Update parameters*: Update the parameters according to the following strategy. Update $\eta _{2,k}$ in the same way as for $n_{k}^{i}$ in Sect. 3 to fit the transition probabilities. Adjust the transfer parameters $\zeta _{j}$ to change the dynamics on the edges of layer two in a homogeneous way (equivalent to changing ${\mathcal{S}}$ in the previous models). If *ζ* is increased, the residence times decrease and the decay rate of the distribution becomes quicker; if *ζ* is decreased, the decay rate is decreased. The residence distribution of the output from layer two is a linear combination of the decay rates governed by *ζ*s. The proportion of each distribution (the mixing) is controlled by the noise on the edges in layer one $\eta _{1,k}$. In the two-node case, for example, if $\eta _{1,1}< \eta _{1,2}$ transitions along $y_{1,2}$ will occur more frequently than along $y_{1,1}$ and so the trajectory will spend longer in node $p_{1,1}$ than $p_{1,2}$.
5. Repeat from 2.

**Figure 8:**
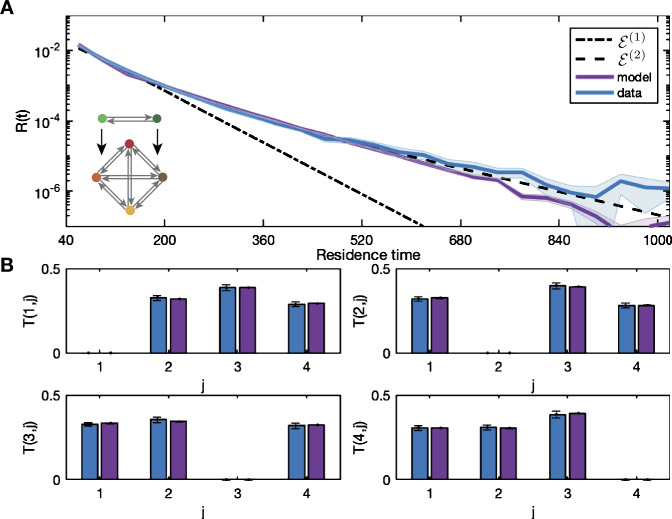
Multi-layer model captures longer residence times. The distribution for the dataset (blue) with the residence time distributions from the simulation of the multi-layer model (purple) with inset network schematic in panel (**A**). The double exponential curve ${\mathcal{E}}^{(2)}$ from Table 1 is also shown. (**B**) shows the transition probabilities. The parameters used in the simulations in each panel are given in Table 4. The model distribution aligns with the two-rate decay of the residence time distribution of the data capturing both long and short times

The results of fitting the multi-layer model with two nodes in layer one to the data are shown in Fig. 8 with the exponential curves ${\mathcal{E}}^{(1)}$ and ${\mathcal{E}}^{(2)}$ from Table 1. The parameters for the simulations and the value of the cost function ${\mathcal{C}}$ are shown in Table 4. The distribution of the simulations agree closely with ${\mathcal{E}}^{(2)}$. Note that the magnitude of the noise on the edges of layer two $\eta _{2,i}$ is much smaller than for the excitable network model. This is due to the additive nature of the transition parameters given by (7).

**Table 4:** Parameters for the multi-layer model. The noise values on the edges $\eta _{l,k}$ for each layer $l=1,2$ with the transfer parameters $\zeta _{n}$ for the model simulation shown in Fig. 8. Cost function ${\mathcal{C}} = 6.993$. Compare $\eta _{2,k}$ to the values in Table 2

Layer-one edges	$\eta _{1,1} $	$\eta _{1,2}$				
	0.04	0.07				
Transfer parameters	$\zeta _{1} $	$\zeta _{2} $				
	0.21	0.0001				
Layer-two edges	$\eta _{2,1}$	$\eta _{2,2}$	$\eta _{2,3}$	$\eta _{2,4}$	$\eta _{2,5}$	$\eta _{2,6}$
	0.0013	0.0020	0.0010	0.0015	0.0023	0.0010
	$\eta _{2,7}$	$\eta _{2,8}$	$\eta _{2,9}$	$\eta _{2,10}$	$\eta _{2,11}$	$\eta _{2,12}$
	0.0013	0.0014	0.0012	0.0013	0.0013	0.0023

In the limit $N \to \infty $ of number of nodes in the controlling layer the distribution would be the sum of infinitely many exponentials, i.e., a power law. However, it is interesting that only two nodes in the controlling layer are sufficient to capture the dynamics. This suggests that the microstate dynamics might be driven by oscillations in other bodily rhythms such as the cardiac or respiratory cycles.

## Comparison of hidden-node and multi-layer network models

So far, we have shown that the hidden-node model and the two-layer model are extensions of the excitable model that both reproduce the transition probabilities and the residence time distributions found in the data. Yet another crucial feature of microstate residence time is that the long and short residence times are interspersed throughout the time-series. For the hidden-node model, the trajectory getting ‘trapped’ at one hidden node should be independent of getting trapped at another hidden node. For the two-layer model, however, the first layer drives the residence times of the second layer, which can lead to clear blocks of short residence times driven by node $p_{1,1}$ with blocks of long residence times driven by node $p_{1,2}$; see, for example, Fig. 7, panel (D).

We now examine the role of the noise weights on the residence times produced by each version of the model. We consider two sets of noise values: exemplar noise values used in Figs. 5 and 7 and the noise values used to fit the models to the data, used in Figs. 6 and 8. The exemplar noise weights on all edges for the hidden-node model are $\eta _{y_{k}}=5\times 10^{-2}$. For the two-layer model, the edge weights are $\eta _{l,k}=10^{-2}$ for $l=1,2$ and all *k*, with transfer parameters $\zeta _{1} = 10^{-1}$ and $\zeta _{2} =10^{-3}$. The noise weights used for the models fitted to the data are shown in Table 3 for the hidden-node model and in Table 4 for the two-layer model.

We assess the generated sequences from the hidden-node and two-layer models by computing the autocorrelation of the signal and the Hurst exponent [37]. Autocorrelation identifies any correlation between the signal and a lagged copy of itself. We compute the autocorrelation by computing the Pearson correlation coefficient between 400 consecutive epochs $\rho (k)$ and $\rho (k+i)$, $k=1,\dots,400$ for 100 lag times $i=1,\dots,100$. The Hurst exponent *H* is a measure of the extent of the LRTC in a time series. For white noise $H=0.5$, whereas $0.5< H<1$ is indicative of LRTC. We compute *H* using wavelet fractal analysis with the code written for [18]. For the data, we use all 24 EEG microstate sequences to compute the Hurst exponent. For each model and set of parameters, we use ten simulations to compute the Hurst exponent.

**Figure 9:**
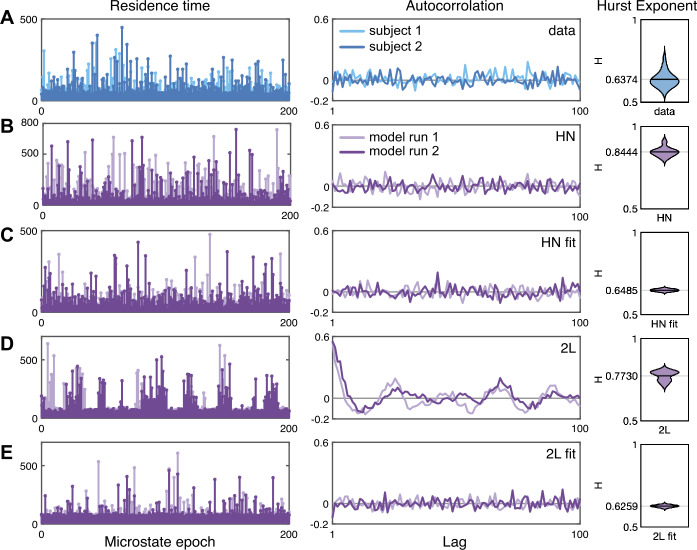
Residence times of 200 consecutive microstate epochs and autocorrelation of 400 microstate epochs from two exemplar subjects and model simulations, with the Hurst exponent computed for all 24 data sets and for 10 simulations of each model. The data is shown in row (**A**). Simulations from the two-layer (2 L) and hidden-node (HN) models using example noise weights on the network edges (see text for details) are shown in rows (**B**) and (**D**). Row (**C**) shows the HN model with noise weights given by Table 3, and row (**E**) shows the 2 L model with noise weights given in Table 4

**Figure 10:**
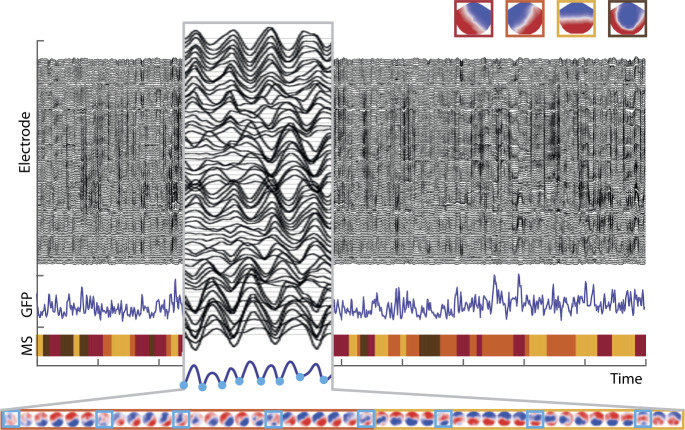
EEG data with backfitting to dominant four microstates. The EEG data and global field potential (GFP) are plotted against time. Each time point of EEG corresponds to a topographic map. The maps corresponding to peaks of GFP are submitted to a clustering analysis that yielded the four dominant template maps shown in the top right. The maps are backfit onto the data in a winner-takes-all process, the result is shown as a color bar where the four colors correspond to the dominant maps. The enlargement shows EEG, GFP, and the topographic maps for each time point. The blue dots mark the local troughs of GFP and the blue boxes mark the corresponding maps. The local troughs of GFP correspond to changes in polarity of the maps

Figure 9 shows the residence times, autocorrelation, and Hurst exponent from the data, the two-layer, and hidden-node network model simulations. In each panel two sequences are shown, for the data these are the durations from two subjects, for the models these are two simulations. The Hurst exponent is shown as a violin plot with the mean value (over all 24 subjects, or over 10 model simulations) marked. The durations from the data show short residence times interspersed with some very long residence times, and the autocorrelation is noisy around zero. The mean Hurst exponent for the data is $H= 0.6374$ as reported in [18] indicating scale-free behavior with LRTC. The hidden-node model with exemplar noise weights has some very large residence times, note that the *y*-axis scale is from 0 to 800 and a mean Hurst exponent of $H=0.8444$. However, when the hidden-node model is fitted to the data, the longest residence times are in line with the data, the autocorrelation is noisy around zero, and $H=0.6485$. The two-layer model residence times with exemplar noise weights show clear blocks alternating between longer residence times and shorter residence times. This is reflected in the oscillatory structure of the autocorrelation and $H=0.7730$. Importantly, fitting the two-layer model to the data abolishes this clustering and renders the long and short residence times interspersed with a corresponding autocorrelation that is again noisy around zero and $H=0.6259$ closer to the value for the data. Finally, we note that the Hurst exponent for sequences generated by the excitable four-node network model (not shown in Fig. 9) is $H\approx 0.5$ indicating no LRTC.

## Discussion

In this article we demonstrate novel mathematical models for EEG microstate sequences constructed using excitable networks that overcome the main caveats of previous modeling approaches that consider microstate sequences as memoryless Markov processes. Resting state brain activity has been shown to visit four canonical EEG microstates with variable durations. The residence times can be considered a residence process that contains interspersed long and short times. Microstate-sequences are mono-fractal and have long range temporal correlations (LRTC) that span two orders of magnitude [18]; the EEG microstate sequences can be thought of as having “memory”. This means that models with memoryless Markov switching between states at times given by a Poisson process will not capture the full temporal dynamics of microstate sequences; more sophisticated modeling approaches are required. Here we demonstrate that excitable network models can capture the crucial features of microstate sequences: the local transition probabilities; the distribution of residence times; the interspersion of long and short residence times; and the LRTC.

We investigate the distribution of residence times of resting state EEG microstate sequences from healthy subjects and show that the distribution has a heavy tail. We show that although the residence time distribution can be fit by a power law, it can also be captured by a sum of exponentials, where each exponential adds a characteristic decay rate to the distribution. We show that a sum of two exponentials is sufficient to capture the heavy tail of the distribution, indicating the data has two characteristic decay rates. We show that the microstate sequences from the data have interspersed long and short residence times giving an autocorrelation of the signal around zero. Finally, we compute the Hurst exponent, a measure of LRTC in time series in line with [18].

We aim to design models that capture the local transitions and residence time distributions observed in the data. To this end, we split each microstate sequence into a local state transition process that gives the probabilities of switching between states and a residence process that produces the timings of microstate transitions. Using the construction outlined in [24], we build systems of stochastic differential equations that each have a noisy network attractor. Specifically, we construct a series of excitable network models and compare these to the data. The simplest, discussed in Sect. 3, simply has four (stable) nodes that represent the four classic microstates. The nodes are all-to-all connected because transitions between any two microstates are possible. Dynamics around the network are driven by noise on the edges, where the level of noise on a given edge governs the probability of transition along that edge. We fit the model to the data by changing the noise on the edges of the network. In this way the model captures the local transition process between microstates. However, this construction produces statistics that are well modeled by a Markov transition process and switching times that are Poisson. In this case the residence time distribution is a single exponential with fast decay rate and no heavy tail. Correspondingly, the Hurst exponent for the simulated sequences is $H\approx 0.5$ indicating no LRTC.

In order to capture the heavy tail of the residence time distribution that appears crucial for the LRTC of the microstate sequences, we extend the single-layer excitable network model using the two following approaches.

First, in Sect. 4, we add one hidden node onto each of the original four nodes, that acts as a ‘trap’ for the trajectories. This creates long residence times at each node. We add two additional noise parameters, one out to the hidden node and one back from it, in order to control the durations of these longer residence times. The local transitions between the original four nodes are unaffected by the hidden nodes and the transition probabilities remain unchanged. With this extension, this model produces residence time distributions with heavy tails, and the simulated microstate sequences show long and short residence times that are interspersed giving an autocorrelation around zero. Moreover, we show that when the model is fitted to the data, simulated sequences have a mean Hurst exponent in line with the data indicating LRTC.

The second extension, in Sect. 5, is the addition of a controlling layer that acts on the edges in the original four-node network, making transitions slower or faster, leading to longer or shorter residence times respectively. The residence time distribution from the data is captured by the sum of two exponentials, therefore two decay rates. Accordingly, we use two nodes in the controlling layer with two corresponding transfer parameters: one that captures the short times and one that captures the long times. The noise on the edges in the controlling layer controls the level of mixing between long and short times in the distribution. When the model is simulated using exemplar noise values and transfer parameters, as shown in Fig. 8, the microstate sequences show clusters of short residence times followed by clusters of long residence times, giving a sinusoidal autocorrelation. The Hurst exponent value of these sequences is higher than the data indicating a smoother trend. When the noise values and transfer parameters of the model are set to produce the correct distribution of residence times, the long and short residence times are interspersed and the autocorrelation decays to noise around zero. Similarly, the Hurst exponent for the fitted sequences is in line with the data. The clustering of long and short times disappears here because the switching between nodes in the controlling layer is faster than the switching between nodes in the original layer when modulated by the slow transfer parameter. Therefore, no transitions occur in the original layer, it remains at one node under the influence of the slow transfer parameter, until the controlling layer switches and the dynamics on the edges in the original layer are modulated by the fast transfer parameter.

The purpose of this paper is to demonstrate that excitable network models can generate sequences that exhibit the features of microstate sequences. The ad-hoc fitting procedure used here demonstrates that these models capture the temporal properties of the switching dynamics more thoroughly than existing statistical models. For further fitting and application of these models to data, a suitable parameter fitting algorithm and systematic parameter space exploration should be employed.

Throughout this paper we use four nodes for the four canonical microstates from [18] and consistently reproduced in the literature [43]. Additional nodes could be added to either of these network constructions to extend the model for larger numbers of microstates, for example, seven [44] or fifteen [45]. The hidden-node network model has $d\times N$ nodes where *d* is the number of decay rates required and *N* is the number of states, whereas the multi-layer model has $d+N$ nodes. Therefore, for larger systems, the multi-layer mode provides a more parsimonious and generalizable modeling approach.

In line with current microstate literature [43, 46] we consider the group level statistics rather than for each individual. We note that while the mean Hurst exponents from the two-layer and hidden-node models align with the data, the spread of the distribution of the Hurst exponent does not. In particular when the models are fitted to the data, the distributions are narrow around the mean; see Fig. 9. The spread of the distribution for the Hurst exponents from the data is likely due to inter-individual variability. We do not fit the models to each individual and do not capture this variability. We leave it to future research to developing the models to explore inter- and intra-individual differences.

These new models support classic microstate analysis and provide insight into the spatial temporal structure of EEG microstate sequences and therefore into the switching dynamics of the underlying neural neural networks. From a neuroscientific perspective, a centrally controlled switching of microstates as in the two-layer model is more parsimonious than a decentralized control of switching as in the hidden-node model. In the former, the switching between the nodes is modulated in the same way for the whole network at once, and in the latter, the switching between nodes happens more independently from one to another. Note that both models capture the data from healthy subjects similarly well. One could consider this difference in locus of switching control as a difference between health and pathology.

Interestingly, neurological and psychiatric conditions rarely affect all microstates similarly, but they generally selectively target only one or two microstates [8, 10–13, 46]. A way to consider the physiological plausibility of the hidden-node model is that altering the connection between a single node-hidden node pair represents a change at the individual microstate level. We plot the individual distributions for each microstate and model with the data in Appendix C. We note that the distributions of the residence times for each microstate produced by the hidden-node and two-layer models give a good fit to the data, as measured by the error values given in Table 5. For the hidden-node model, the noise values governing frequency of visits to the hidden nodes ($y_{\mathrm{out}}$) and duration spent there ($y_{\mathrm{in}}$) are the same for all nodes. Changing the $y_{\mathrm{in}}$ and $y_{\mathrm{out}}$ values allows adaptation of the hidden-node model to describe differences between healthy and pathological groups.

**Table 5:** The error *χ* computed for between the mean curves from the model and the data for each microstate shown in Fig. 11

Data compared to	MS 1	MS 2	MS 3	MS 4
one-layer fit	26.896	41.970	105.915	49.978
hidden-node fit	19.681	5.694	0.978	6.071
two-layer fit	15.778	4.914	40.274	6.717

The modeling techniques described in this paper are widely applicable to other physiological and synthetic signals. For example, in task situations, the microstate immediately before a stimulus has been shown to predict stochastically occurring variations in the perceptual fate of upcoming stimuli; this pertains to hemispheric lateralization of word processing, perceptual switching of multi-stable stimuli, and perceptual awareness at the sensory threshold [47–51]. The models presented here, in particular the two-layer model, could be extended and used to investigate the relationship between brain activity and perception.

A way to consider the physiological plausibility of the two-layer model would be to relate EEG microstates to other bodily rhythms such as the cardiac or respiratory cycle. The cardiac cycle can be subdivided into the systole (contraction of the heart muscles) and diastole (relaxation of the heart muscles). The systole is shorter and less variable than the diastole, and they could be likened to the two nodes in the controlling layer of the two-layer model. Similarly, brain activity (local field potential (LFP) activity in limbic networks implied in memory formation) is tightly coupled to the respiratory cycle both in rodents and humans [52–54]. Hence, simultaneous recordings of EEG, electrocardiogram (ECG), and respiration could provide an interesting way of testing the physiological plausibility of the two-layer model by relating the different layers to different physiological processes.

Future research could pit the performance of the different models against each other by applying them to data from patients with pathologies of different aetiologies: while the hidden-node model should better capture the changes due to psychiatric diseases that affect only one or two microstates, the two-layer model should capture potential differences in microstate sequences in patients with pathologies affecting, for example, their cardiac rhythm.

## Figures and Tables

**Figure 11:**
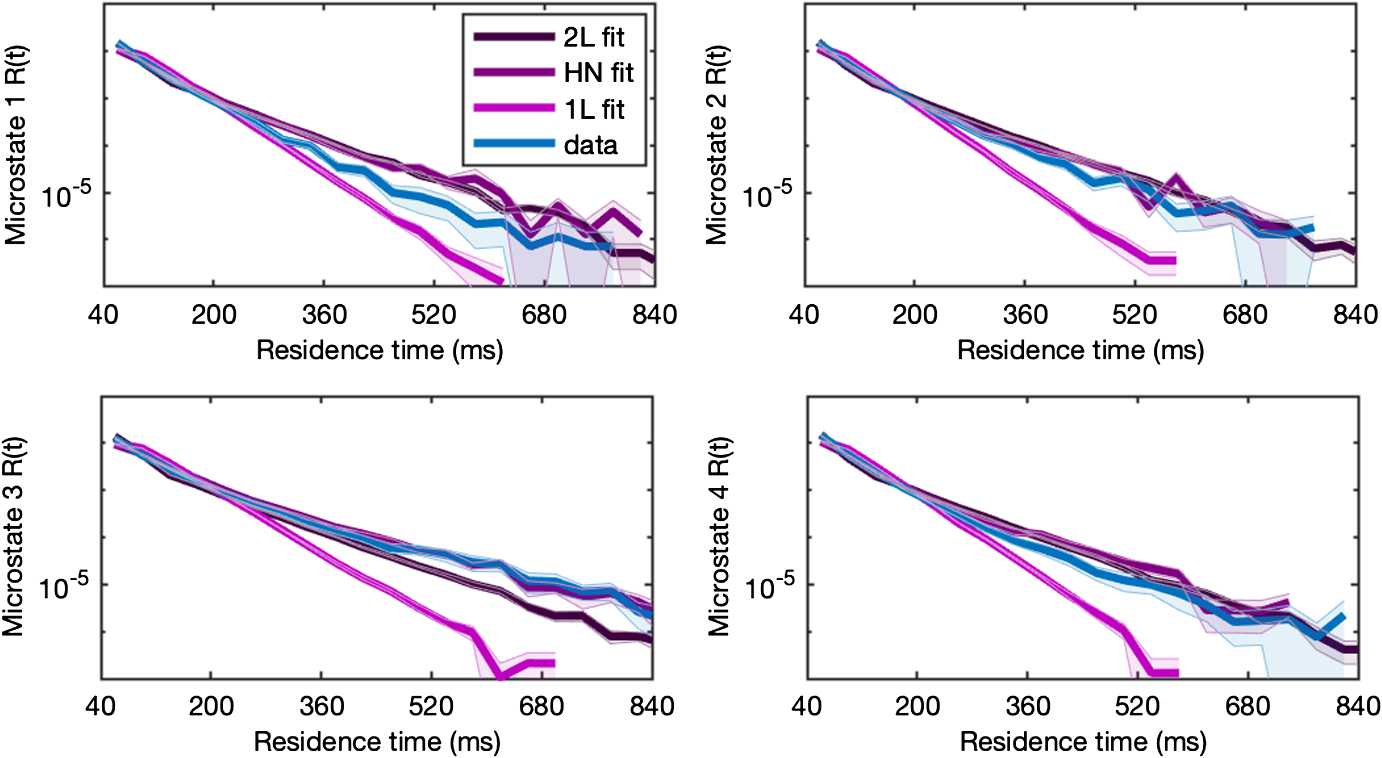
Residence time distributions for each microstate from the data and each model. The model simulations after the fitting procedure are used. The least squares distances between the distributions are shown in Table 5. In line with the main results, the residence distributions of the one-layer model do not capture the heavy tails for any microstate, whereas the two-layer and hidden-node models show a much better fit even though the models are not fitted to the distributions of the individual microstates

## Data Availability

The data used in this paper are published in [18] and are available from JB on reasonable request.

## Appendix A: Illustration of microstate back-fitting procedure

Microstate analysis comprises two steps: identifying the microstate template maps (cluster analysis) and determining the microstate parameters underlying their strength and timing (back-fitting). The latter comprises a time-point wise spatial correlation between the template maps identified in the cluster analysis and the continuous data, and labels are assigned in a winner-takes-all fashion, i.e., the template that correlates highest with the data is assigned at that time point. At the local troughs of GFP, where the field strength is minimal and the global dissimilarity is high, none of the template maps correlate well with the data, and the allocation of the best template can be considered random. Without applying a temporal constraint criterion, these brief periods will be accounted as very short separate microstates, and microstates could never be longer as the period between the local GFP troughs. In other words, a temporal constraint criterion is necessary in order to ‘jump across’ the GFP troughs and ignore the polarity. Without this temporal constraint, one would obtain a peak around 2–3 sampling periods corresponding to the local GFP troughs and corresponding to 16–24 ms (at a sampling rate of 125 Hz) in our case.

Figure 10 shows and example series of maps from consecutive times points of the EEG. The maps corresponding to troughs in GFP are marked. The first 24 time point maps were assigned to microstate map 2 (orange) and the remaining time point maps to microstate 3 (yellow). The temporal constraint ensures that the toughs within each period are not assigned to different microstates. It is important to note that microstates are characterized by topography alone, i.e., the polarity of the topography is irrelevant for characterizing a microstate, and a given microstate can undergo several polarity changes, which occur at the local troughs of GFP. Note that each microstate period undergoes several polarity changes.

## Appendix B: Network models with heteroclinic/excitable networks

We briefly specify the dynamical models used to flexibly construct heteroclinic or excitable networks with arbitrary transition probabilities and waiting times. The models, and why they robustly have attracting networks of connections between equilibria in phase space, are detailed in [24].

### B.1 Single-layer network model

We use the following functions to define the network dynamic model presented in (3, 4), and for the generalization to include additional hidden nodes:

8 $$\begin{aligned} &f(p_{j},y_{k}) = p_{j} \bigl(F \bigl(1- p^{2} \bigr)+ D \bigl(p_{j}^{2}p^{2}-p^{4} \bigr) \bigr)+ E \bigl(-Z^{(O)}_{j}(p,y)+Z^{(I)}_{j}(p,y) \bigr), \end{aligned}$$

9 $$\begin{aligned} &g(p_{j},y_{k}) = -y_{k} \bigl( \bigl(y_{k}^{2}-1\bigr)^{2}+ A-B p_{\alpha (k)}^{2} +C \bigl(y^{2}-y_{k}^{2} \bigr) \bigr), \end{aligned}$$

where $j=1,\ldots,M$, $k=1,\ldots,Q$ and

$$ p^{2}=\sum_{j=1}^{M} p_{j}^{2},\qquad p^{4}=\sum _{j=1}^{M} p_{j}^{4},\qquad y^{2}=\sum_{j=1}^{Q} y_{j}^{2}. $$

The outputs $(O)$ and inputs $(I)$ to the *p* cells from the *y* cells are determined by

10 $$\begin{aligned} &Z^{(O)}_{j}(p,y) = \sum_{\{k: \alpha (k)=j\}} y_{k}^{2}p_{\omega (k)}p_{j}, \end{aligned}$$

11 $$\begin{aligned} &Z^{(I)}_{j}(p,y) = \sum_{\{k': \omega (k')=j\}} y_{k'}^{2}p_{ \alpha (k')}^{2}. \end{aligned}$$

To ensure the network is in the excitable regime, we fix the constants in functions *f* and *g* throughout as follows:

$$ A=0.5,\qquad B=1.49,\qquad C=2,\qquad D=10, \qquad E=4, \qquad F=2 $$

although the behavior is robust to small enough changes to these parameters. See [24] for more details and justification of how the model exhibits an excitable network where trajectories explore a set of *N* nodes along *M* possible directed edges.

### B.2 Multi-layer network model

The functions *f* and *g* in the multi-layer model (6, 7) are defined by

12 $$\begin{aligned} &f_{l}(p_{l,j},y_{l,k}) = p_{l,j} \bigl(F\bigl(1- p_{l}^{2}\bigr)+ D\bigl(p_{l,j}^{2}p_{l}^{2}-p_{l}^{4} \bigr) \bigr) + E \bigl(-Z^{(O)}_{l,j}(p,y)+Z^{(I)}_{l,j}(p,y) \bigr), \end{aligned}$$

13 $$\begin{aligned} &g_{l}(p_{l,j},y_{l,k}) = -y_{l,k} \bigl(\bigl(y_{l,k}^{2}-1\bigr)^{2}+A - B p_{l, \alpha (k)}^{2} +C\bigl(y_{l}^{2}-y_{l,k}^{2} \bigr) \bigr) \end{aligned}$$

for $j=1,\dots,M_{l}$ and $k=1,\dots,Q_{l}$. The outputs $(O)$ and inputs $(I)$ to the *p* cells from the *y* cells are as follows:

14 $$\begin{aligned} &Z^{(O)}_{l,j}(p,y) = \sum_{\{k: \alpha (k)=j\}} y_{l,k}^{2}p_{l, \omega (k)}p_{l,j}, \end{aligned}$$

15 $$\begin{aligned} &Z^{(I)}_{l,j}(p,y) = \sum_{\{k': \omega (k')=j\}} y_{l,k'}^{2}p_{l, \alpha (k')}^{2}, \end{aligned}$$

and

$$ p_{l}^{2}=\sum_{j=1}^{M_{l}} p_{l,j}^{2},\qquad p_{l}^{4}=\sum _{j=1}^{M_{l}} p_{l,j}^{4},\qquad y_{l}^{2}=\sum_{k=1}^{Q_{l}} y_{l,k}^{2}. $$

The default parameters *A*–*F* are as in Appendix B.1.

## Appendix C: Individual microstate distributions

The individual microstate distributions for each model are shown in Fig. 11. The error *χ* is the least squares difference between the log of the values of each curve and the log of the data and the measure of fit, given in Table 5. In line with the main results, the one layer model does not capture the distributions of the individual microstates and the errors *χ* are large. The distributions of the two-layer and hidden-node models have a smaller error $\chi <20$ for all microstates. The agreement shown here further demonstrates the potential for this model to capture properties of interest in microstate analysis.

## Abbreviations

EEG: Electroencephalogram
MRI: Magnetic resonance imaging
fMRI: Functional magnetic resonance imaging
RSNs: Resting-state networks
LRTC: Long-range temporal correlations
BOLD: Blood-oxygen-level-dependent
DFA: Detrended fluctuation analysis
SDEs: Stochastic differential equations
GFP: Global field power
AAHC: Atomize-agglomerate hierarchical clustering
LFP: Local field potential
ECG: Electrocardiogram
